# Third intracellular loop of HCMV US28 is necessary for signaling and viral reactivation

**DOI:** 10.1128/jvi.01801-24

**Published:** 2024-12-10

**Authors:** Samuel Medica, Michael Denton, Nicole L. Diggins, Olivia Kramer-Hansen, Lindsey B. Crawford, Adam T. Mayo, Wilma D. Perez, Michael A. Daily, Christopher J. Parkins, Luke E. Slind, Lydia J. Pung, Whitney C. Weber, Hannah K. Jaeger, Zachary J. Streblow, Gauthami Sulgey, Craig N. Kreklywich, Timothy Alexander, Mette M. Rosenkilde, Patrizia Caposio, Meaghan H. Hancock, Daniel N. Streblow

**Affiliations:** 1 Vaccine and Gene Therapy Institute, Oregon Health & Science University 56870 , Beaverton, Oregon, USA; 2 Department of Molecular Microbiology and Immunology, Oregon Health and Science University 547642 , Portland, Oregon, USA; 3 Department of Biomedical Sciences Molecular Pharmacology, University of Copenhagen 4321 , Copenhagen, Denmark; 4 Division of Pathobiology and Immunology, Oregon National Primate Research Center 88960 , Beaverton, Oregon, USA; Northwestern University Feinberg School of Medicine, Chicago, Illinois, USA

**Keywords:** cytomegalovirus, latency, reactivation, G-protein-coupled receptor, signal transduction

## Abstract

The human cytomegalovirus (HCMV) encoded chemokine receptor US28 plays a critical role in viral pathogenesis, mediating several processes such as cellular migration, differentiation, transformation, and viral latency and reactivation. Despite significant research examining the signal transduction pathways utilized by US28, the precise mechanism by which US28 activates these pathways remains unclear. We performed a mutational analysis of US28 to identify signaling domains that are critical for functional activities. Our results indicate that specific residues within the third intracellular loop (ICL3) of US28 are major determinants of G-protein coupling and downstream signaling activity. Alanine substitutions at positions S218, K223, and R225 attenuated US28-mediated activation of MAPK and RhoA signal transduction pathways. Furthermore, we show that mutations at positions S218, K223, or R225 result in impaired coupling to multiple Gα isoforms. However, these substitutions did not affect US28 plasma membrane localization or the receptor internalization rate. Utilizing CD34^+^ HPC models, we demonstrate that attenuation of US28 signaling *via* mutation of residues within the ICL3 region results in an inability of the virus to efficiently reactivate from latency. These results were recapitulated *in vivo*, utilizing a humanized mouse model of HCMV infection. Together, our results provide new insights into the mechanism by which US28 manipulates host signaling networks to mediate viral latency and reactivation. The results reported here will guide the development of targeted therapies to prevent HCMV-associated disease.

**IMPORTANCE:**

Human cytomegalovirus (HCMV) is a β-herpesvirus that infects between 44% and 100% of the world population. Primary infection is typically asymptomatic and results in the establishment of latent infection within CD34^+^hematopoietic progenitor cells (HPCs). However, reactivation from latent infection remains a significant cause of morbidity and mortality in immunocompromised individuals. The viral chemokine receptor US28 influences various cellular processes crucial for viral latency and reactivation, yet the precise mechanism by which US28 functions remains unclear. Through mutational analysis, we identified key residues within the third intracellular loop (ICL3) of US28 that govern G-protein coupling, downstream signaling, and viral reactivation *in vitro* and *in vivo*. These findings offer novel insights into how US28 manipulates host signaling networks to regulate HCMV latency and reactivation and expand our understanding of HCMV pathogenesis.

## INTRODUCTION

Human cytomegalovirus (HCMV) is an opportunistic pathogen that infects a large proportion of the world’s population (1). HCMV remains the most common congenital infection and causes severe neurological and developmental deficits in neonates (2). Furthermore, HCMV infection is a significant cause of morbidity and mortality in organ transplant recipients and otherwise immunocompromised populations (3). Primary infection with HCMV is typically asymptomatic and results in the establishment of viral latency within CD34^+^ hematopoietic progenitor cell (HPC) and CD14^+^ monocyte reservoirs (4). During latent infection, the HCMV genome exists in a quiescent state limiting gene expression to evade detection by the host immune system (5). During viral reactivation, differentiated macrophages and dendritic cells generated from these reservoirs disseminate the virus throughout the host (6, 7). Despite significant research examining the cues governing the switch from latent to lytic infection, the specific molecular mechanisms mediating these processes remain unclear. To date, no FDA-approved vaccine against HCMV exists and current antiviral regimens often have severe adverse side effects (8–10). Moreover, these therapeutic agents only act during lytic infection when clinical manifestations are already present and promote the evolution of drug-resistant HCMV strains. Therefore, to facilitate the advancement of new therapies targeting HCMV, it is imperative to clarify the precise cellular and viral mechanisms responsible for mediating HCMV latency and reactivation.

G-protein-coupled receptors (GPCRs) represent the most abundant and diverse family of membrane-bound proteins in the human genome and are targets for over one-third of FDA-approved pharmaceuticals (11). GPCRs are characterized as containing an extracellular N-terminal domain, seven alpha-helical transmembrane domains, and an intracellular C-terminal domain (12, 13). Extracellular loops formed by the transmembrane domains assist with facilitating ligand binding while the intracellular loops, primarily intracellular loops two and three (ICL2/3), are paramount for G-protein coupling and selectivity (14, 15). Upon interfacing with extracellular ligands, GPCRs undergo conformational changes allowing them to couple with cognate heterotrimeric G-protein complexes consisting of α-, β-, and γ-subunits. GPCRs act as guanine nucleotide exchange factors (GEFs) catalyzing the exchange of GDP for GTP on the Gα subunit. Once bound to GTP, the α-subunit dissociates from the βγ-subunits to activate downstream signal transduction effectors and ultimately alter gene expression. The exact cellular signal transduction cascades initiated by G-proteins are dependent on cell type, GPCR class, ligand specificity, and activated Gα isoform. Gα proteins are broadly organized into four families: Gα_s_, Gα_i/o_, Gα_q/11_, and Gα_12/13_ based on their structural composition and signaling characteristics (12, 16). Together, GPCR–G-protein interactions, and downstream signaling, are responsible for modifying nearly all facets of cellular processes relating to metabolism, cellular migration, proliferation, and cytoskeletal remodeling.

HCMV encodes four putative GPCRs including US28, which exhibits homology to the host CC-chemokine receptors CCR1 and CCR5 (17, 18). US28 is expressed with early kinetics and is packaged into the mature virion (19, 20). Within infected cells, US28 is primarily localized to intracellular multivesicular bodies and is continuously internalized from the cell surface *via* both β-arrestin and clathrin-mediated mechanisms (21–24). US28 binds both CC and CX_3_C chemokines and is capable of coupling to multiple Gα isoforms including Gα_q/11_, Gα_i/o_, and Gα_12/13_ subfamilies (18, 25–28). Dependent on the infected cell type and interacting G-protein, US28 is capable of interfacing with several canonical signal transduction pathways to activate transcription factors such as NFAT, CREB, NF-κB, ELK/SRE, STAT3, SRF, and TCF/LEF (29–34). For instance, in arterial smooth muscle cells, binding of CCL2 or CCL5 to US28 stimulates cellular migration, whereas binding of CX_3_CL1 blocks this effect (27). A reverse of this ligand-specific phenotype was observed in macrophages wherein CX_3_CL1 promoted migration and CC ligands blocked it, highlighting the cell type-specific effector functions of US28. Within CD34^+^ HPCs, US28 drives cellular differentiation down the myeloid lineage through an undefined mechanism (35). Importantly, US28 is expressed during both latent and lytic phases of the viral lifecycle, and ligand-dependent signaling is essential for the establishment of latent infection and viral reactivation; however, the exact mechanisms by which US28 facilitates these processes remain unclear (35–39).

In the present study, we conducted a mutational analysis of US28 aimed at identifying crucial motifs essential for signaling and functional capabilities. Our findings reveal that specific residues within the US28 ICL3 play a pivotal role in determining G-protein coupling and downstream signaling activity. Notably, mutations at positions S218, K223, and R225 led to diminished US28-mediated activation of mitogen-activated protein (MAP) kinase and RhoA signal transduction. Moreover, we observed that alanine substitution at positions S218, K223, and R225 significantly impaired the coupling of US28 to multiple Gα isoforms, while having no impact on its plasma membrane localization or internalization kinetics. Furthermore, we illustrate that the attenuation of US28 G-protein coupling and downstream signaling hampers the virus’s ability to efficiently reactivate in CD34^+^ HPCs. Importantly, these findings were replicated *in vivo* using a humanized mouse model of HCMV infection. Collectively, our results contribute novel insights into the mechanism through which US28 influences host signaling networks to orchestrate the complex interplay between latent and lytic infection. The findings of this study will be integral for the development of novel therapeutics preventing HCMV-associated disease.

## RESULTS

### Activation of Gα_q/11_ and downstream signal transduction effectors is required for efficient viral reactivation

Previous studies have shown that US28 is capable of activating the Gα_q/11_ family of G-proteins to stimulate several signal transduction cascades such as MAPK, NF-κB, and PLC-β (32, 40, 41). Importantly, many of these signal transduction pathways play an integral role in the establishment of viral latency or the capacity to reactivate (42–46). To understand how the activation of Gα_q/11_ influences viral latency and reactivation, we identified YM-254890, a selective inhibitor of the Gα_q/11_ family of G-proteins (47). Treatment with YM-254890 in primary human fibroblasts showed minimal cytotoxic effects at 72 hours post-treatment at concentrations up to 10 µM (Fig. S1A). Moreover, treatment of HCMV-infected fibroblasts with YM-254890 exhibited no discernable effects on lytic viral replication as measured *via* nano-luciferase (nLuc) reporter assay and by limiting dilution plaque assays (Fig. S1B and C). To evaluate the effects of YM-254890 on US28 signaling activity, we performed luciferase reporter assays in transiently transfected HEK-293 cells monitoring US28-mediated activation of MAPK signaling. Treatment with YM-254890 resulted in dose-dependent inhibition of US28-mediated activation of the SRE reporter element (Fig. S1D) suggesting that inhibiting US28– Gα_q/11_ signaling blocks US28-mediated MAPK activation.

**Fig 1:**
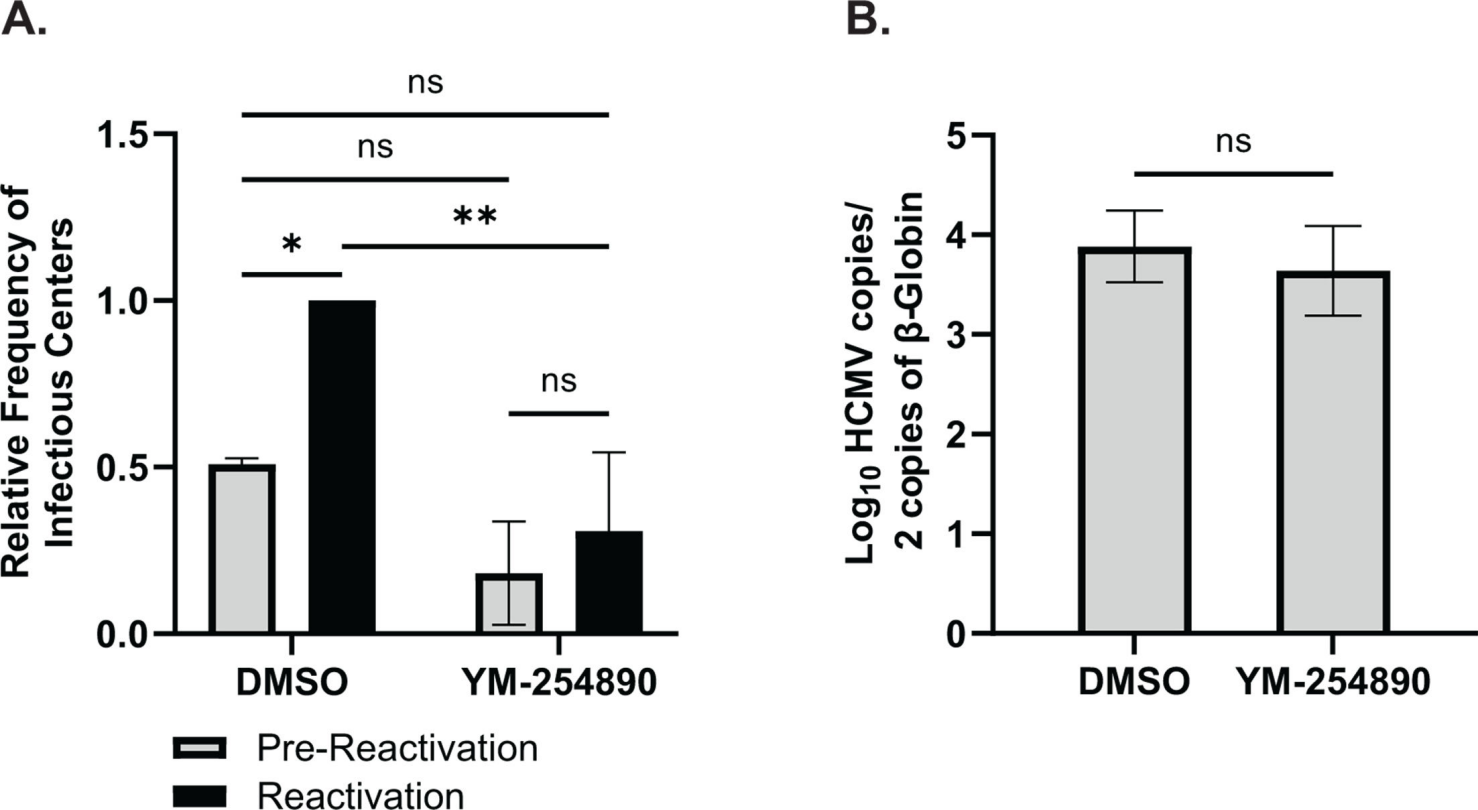
Activation of Gα_q/11_ is required for efficient reactivation. hESC-derived CD34^+^ HPCs were infected with TB40/E-GFP at a multiplicity of infection (MOI) of 2 for 48 hours. Cells were FACS isolated for viable CD34^+^/GFP^+^ HPCs and were cultured above a murine stromal cell support layer for 12 days to establish latent infection. Cells were treated with 1 µM YM-254890 (Gα_q/11_ inhibitor) or an equivalent amount of DMSO throughout the latency culture. (**A**) At 14-DPI, half of the cells were treated with a reactivation cocktail and plated onto a fibroblast monolayer, the other half of the cells were lysed and used to infect fibroblasts directly (pre-reactivation control). Reactivation was assessed by the frequency of infectious centers as determined *via* ELDA (51, 52) at 3 weeks post-plating. Data are shown as fold change in infectious centers, as compared to the reactivation group, for triplicate experiments. Error bars represent the standard error of the mean and statistical significance was calculated using two-way ANOVA followed by Tukey’s post hoc analysis (*P < 0.05/**P < 0.01). (**B**) At 14-DPI, total DNA was harvested from infected CD34^+^ HPCs, and viral genomes were quantified *via* qPCR using primers and probes specific for the viral UL141 gene. Viral genomes were normalized to total cell number using human β-globin as a reference gene. Data represent the mean Log_10_ transformed values for triplicate experiments. Error bars represent the standard error of the mean and statistical significance was calculated using a student’s t-test.

To determine whether Gα_q/11_ activation affects the establishment of viral latency or the capability of the virus to reactivate, we infected human embryonic stem cell (hESC)-derived CD34^+^ HPCs with HCMV TB40/E-GFP (48, 49). Infected CD34^+^ HPCs were isolated *via* FACS and cultured above a murine stromal support layer under conditions that favor latent infection. At the time of initial plating, the cell culture media was supplemented with 1 µM of YM-254890 or an equivalent amount of vehicle (DMSO). At 14 days post-infection (DPI), half of the HPCs were lysed (pre-reactivation), and the remaining cells were treated with granulocyte-macrophage colony-stimulating factor (GM-CSF) and granulocyte colony-stimulating factor (G-CSF) to induce cellular differentiation and viral reactivation. Cells and lysates were plated over a monolayer of fibroblasts to perform an extreme limiting dilution assay (ELDA) assessing the frequency of infectious centers up to 3 weeks post-plating (50). Comparable levels of virus were present in YM-254890-treated versus vehicle-treated latently infected cells (pre-reactivation) indicating that Gα_q/11_ signal transduction has minimal effect on the establishment of viral latency (Fig. 1A; Fig. S2). By contrast, treatment with YM-254890 resulted in a significant decrease in the ability of the virus to efficiently reactivate (Fig. 1A; Fig. S2). Because an observed deficit in viral reactivation can be due to an inability to maintain viral genomes throughout the latency establishment period, we quantified HCMV genomes at the end of latent infection *via* quantitative PCR. Results from these experiments confirmed that inhibition of Gα_q/11_ signal transduction causes a true reactivation deficit since a comparable number of viral genomes were present in YM-254890 and DMSO-treated cells (Fig. 1B). Together, these results indicate that activation of Gα_q/11_, through US28 or other mechanisms, is required for efficient HCMV reactivation in CD34^+^ HPCs.

### Mutations in the third intracellular loop of US28 decrease signaling activities despite maintaining similar localization and internalization

**Fig 2:**
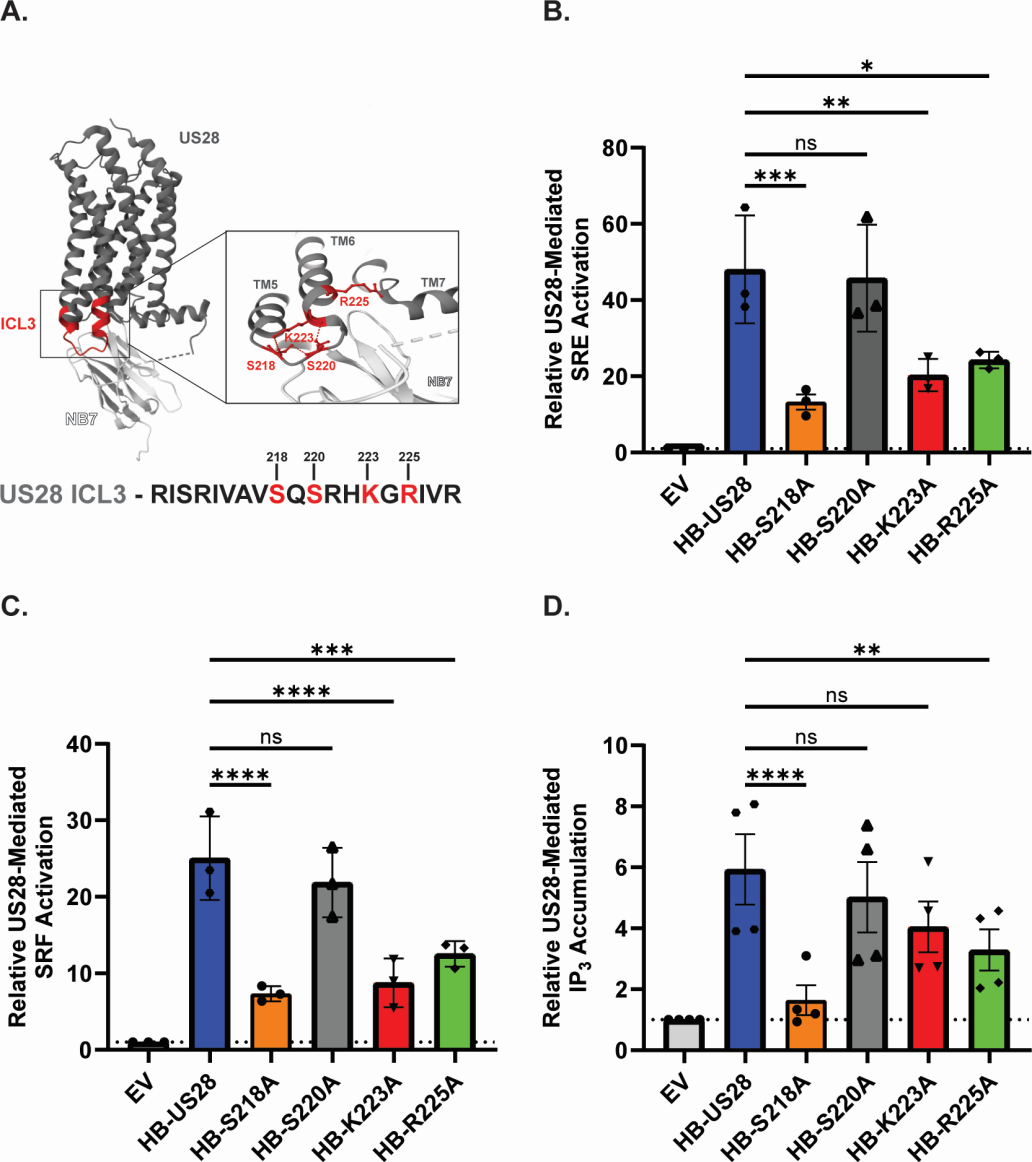
Mutational analysis of the US28 ICL3 identifies residues required for signaling activity. (**A**) Structural representation of the third intracellular loop of US28 with alanine substituted residues denoted in red. Adapted from (62) (B and C) HEK-293 cells were transfected with the indicated constructs along with Renilla and Firefly luciferase reporter plasmids for SRE (**B**) or SRF (**C**). At 18 hours post-transfection, media was exchanged with serum-free DMEM. Luciferase activity was measured using the Dual-Luciferase Reporter Assay System (Promega) at 6 hours post-media exchange. Data are shown as fold change relative to transfection with the empty vector for triplicate experiments. Error bars represent the standard error of the mean. Statistical significance was calculated by one-way ANOVA followed by Dunnett’s multiple comparison post hoc analysis (*P < 0.05, **P < 0.01, ***P < 0.001/****P < 0.0001*).* (**D**) HEK-293 cells were transfected with the indicated constructs or the empty vector (EV) and incubated with tritium labeled myo-inositol. At 48 hours post-transfection, cells were washed and incubated with LiCl prior to cell lysis. Lysates were incubated with SPA-YSi beads for 8 hours and scintillation was measured. Data are plotted as the fold change relative to transfection with the empty vector for quadruplicate experiments. Error bars represent the standard error of the mean. Statistical significance was calculated by one-way ANOVA followed by Dunnett’s multiple comparison post hoc analysis (**P < 0.01/****P < 0.0001)*.*

**Fig 3:**
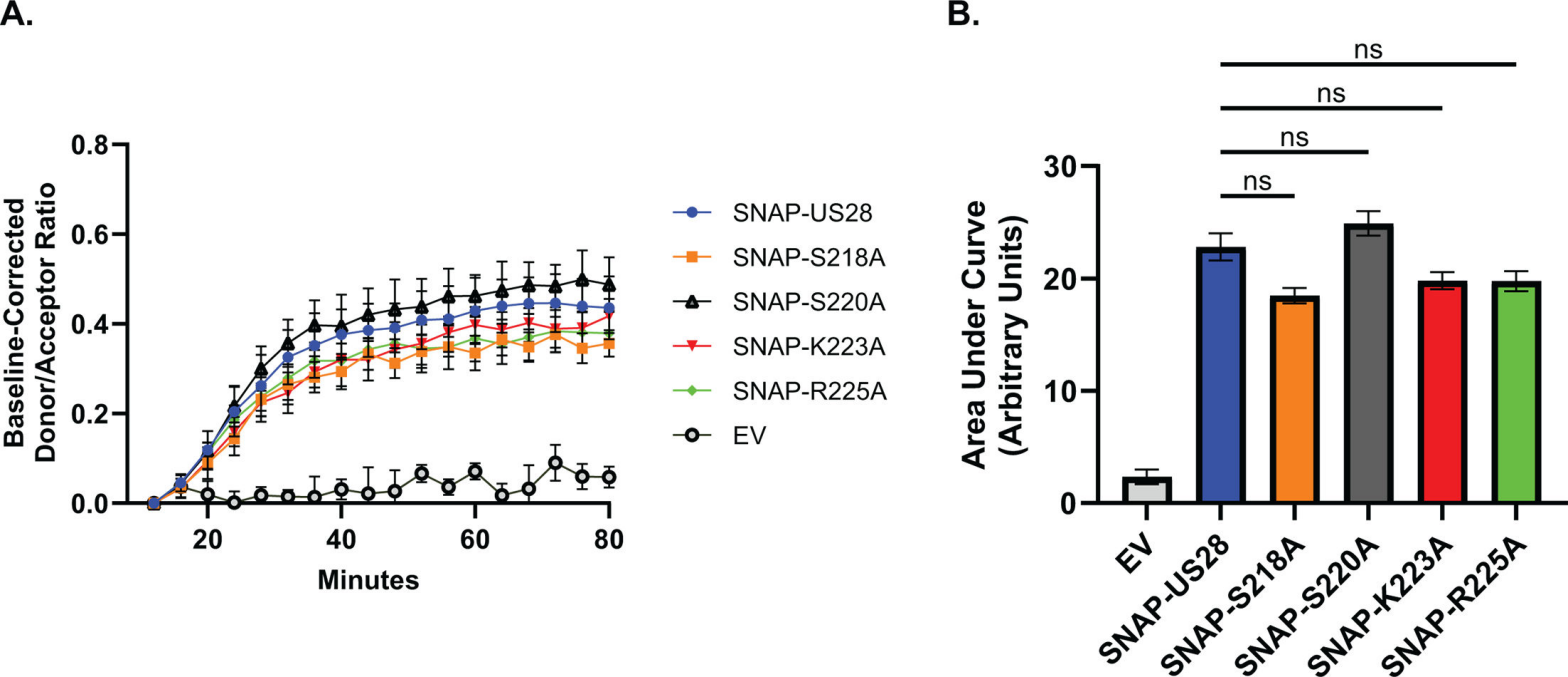
Mutations in the ICL3 region of US28 do not affect receptor internalization kinetics. (**A**) HEK-293 cells were transfected with the indicated SNAP-US28 constructs or the empty vector (EV). At 48 hours post-transfection, cells were treated with SNAP-Lumi4-Tb (donor) for 1 hour prior to washing. Fluorescein (acceptor) was added to the appropriate wells and internalization kinetics were measured for 80 minutes. Data are plotted as the baseline corrected donor/acceptor ratio (abs 615/520 nm). Error bars represent the standard error of the mean for triplicate experiments. (**B**) The area under the curve was calculated and plotted for each profile. Error bars represent the standard error of the mean between triplicate experiments. Statistical significance was calculated using one-way ANOVA followed by Dunnett’s multiple comparison post hoc analysis.

Next, we sought to identify regions of US28 that are required for propagating downstream signaling activity. Because the third intracellular loop (ICL3) between transmembrane domains five and six is a major determinant of G-protein coupling in other class A GPCRs (53–58), we generated alanine substitutions for basic and polar residues within this region (S218, S220, K223, and R225). The wild-type and ICL3 mutant constructs contained an in-frame N′ terminal 11 amino acid HiBiT tag to quantify protein production and surface expression (Fig. 2A). Equivalent expression and membrane localization of each mutant construct was verified by immunoblot and HiBiT detection assay in transiently transfected HEK-293 cells (Fig. S3). To examine the effects of these mutations on known US28 signal transduction pathways, we conducted luciferase reporter assays monitoring US28-mediated activation of MAPK and RhoA signaling (39, 59, 60). Alanine substitution at positions S218, K223, and R225 significantly reduced US28-mediated activation of MAPK as indicated by a reduction in luminescence driven by the SRE reporter element (Fig. 2B). In a similar manner, the same three mutants exhibited decreased US28-mediated activation of RhoA as indicated by a reduction in luminescence driven by the SRF reporter element (Fig. 2C). No significant effect was observed regarding alanine substitution at position S220 for either MAPK or RhoA signal transduction (Fig. 2B and C). Results from these experiments were further confirmed by immunoblot monitoring ERK phosphorylation and RhoA activation (Fig. S4). Because US28 signals through phospholipase C (PLC), we performed scintillation proximity assays to monitor the accumulation of the downstream signaling intermediary inositol triphosphate (IP_3_) for the US28 mutants (61). Interestingly, results from these experiments showed that only alanine substitutions at S218 and R225 showed a significant reduction (Fig. 2D). Because a decrease in signaling activity could be associated with aberrations in receptor internalization, we monitored the real-time internalization kinetics for each of our constructs *via* a FRET-based assay. To this end, we engineered an in-frame SNAP-tag on the N’ terminus of the wild-type US28 and the ICL3 mutant receptors. In this assay, the SNAP tag serves as the energy donor and exogenous fluorescein as the energy acceptor. Following optimization of the transfection conditions to achieve a comparable donor signal for each of the constructs (Fig. S5), the real-time internalization experimental data show that mutations in the ICL3 region do not alter the internalization kinetics of US28 (Fig. 3). Together, these findings suggest that specific residues within the ICL3 region of US28 are required for signal transduction but not for receptor localization or internalization.

### Third intracellular loop of US28 is necessary for G-Protein coupling

**Fig 4:**
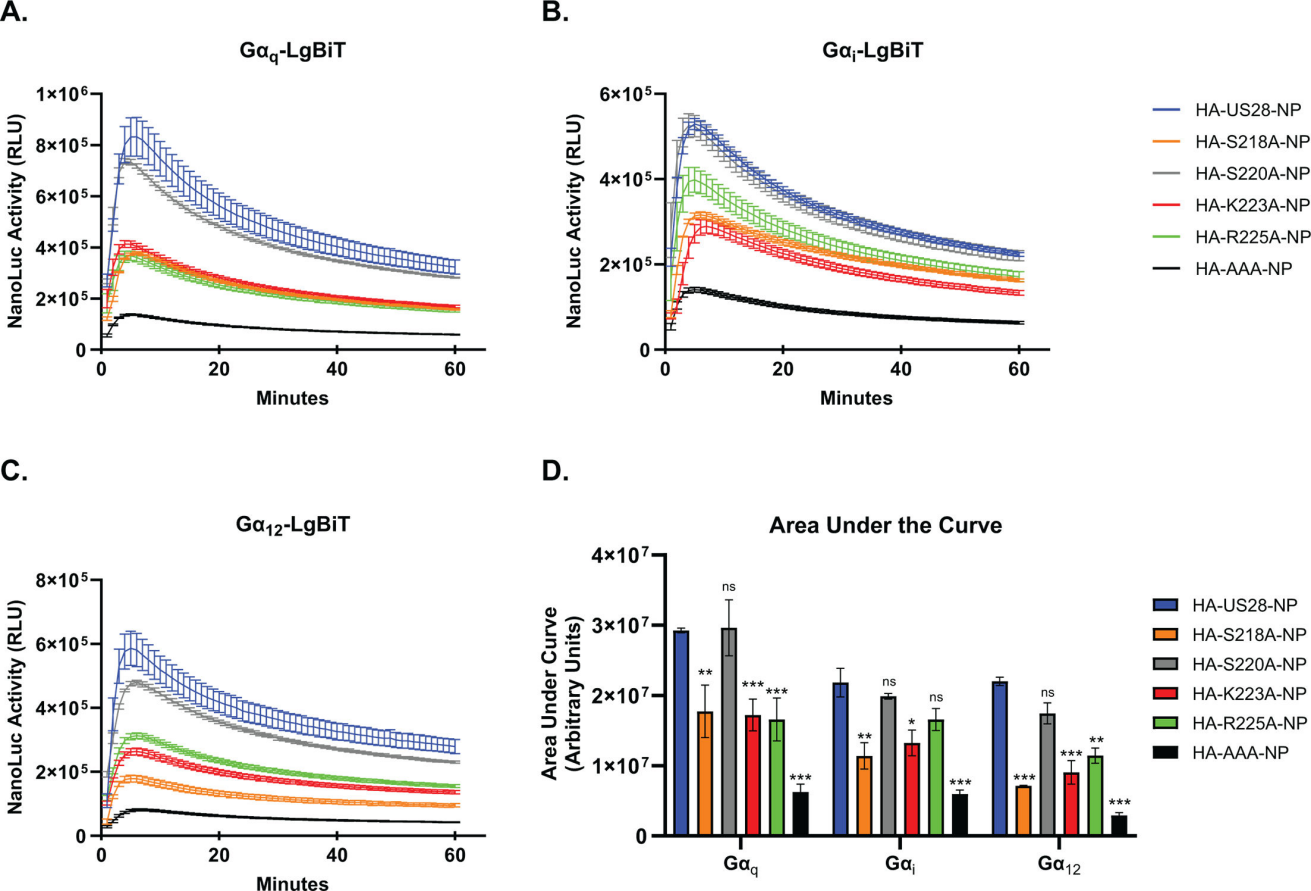
US28 ICL3 mutants exhibit impaired G-protein coupling. HEK-293 cells were transfected with the indicated natural peptide-tagged US28 constructs or the empty vector (EV) and LgBiT tagged (**A**) Gα_q_, (**B**) Gα_i_, or (**C**) Gα_12_. At 18 hours post-transfection, media was exchanged with serum-free DMEM. At 6 hours post-media replacement, luciferase activity was monitored for 60 minutes using the Nano-Glo live cell assay system (Promega). Error bars represent the standard error of the mean between technical triplicates. (**D**) The area under the curve was calculated for each profile and plotted. Error bars represent the standard error of the mean between triplicate experiments. Statistical significance was calculated using two-way ANOVA followed by Dunnett’s multiple comparison post hoc analysis (*P < 0.05/**P < 0.01/***P < 0.001)*.*

To further understand the reduction in US28 signaling activity observed with mutation at residues S218, K223, and R225, we assessed the ability of our mutant constructs to efficiently couple with heterotrimeric G-protein complexes. To this end, we made use of a nLuc-based complementation assay measuring real-time interactions that was described previously by Laschet and colleagues (63). In this system, the C′ terminus of the GPCR is linked in frame to one of three NanoBiT subunits (HiBiT, SmBiT, or the native natural peptide [NP]) while the Gα subunit is genetically fused to the complementing Large Bit (LgBiT) (63). We chose to use natural peptide as our NanoBiT as it provides an optimal balance between interaction affinity/reversibility and strength of signal (63). Consequently, we engineered an in-frame natural peptide tag on the C′ terminus of the wild-type US28 receptor followed by site-directed mutagenesis substituting alanine at positions S218, S220, K223, and R225. As a negative control, we generated a construct with substitutions in the canonical G-protein-coupling domain of US28 (DRY_128-130_–AAA_128-130_) located at the base of TM3. Mutations within this region prevent US28 coupling to respective heterotrimeric G-protein complexes rendering the receptor “signaling dead” (18, 31, 37, 64, 65). Equivalent expression of each construct was verified by immunoblot in transiently transfected HEK-293 cells (Fig. S6). In live cell GPCR interaction assays with LgBiT-Gα_i1_, -Gα_q_, and -Gα_12_, US28 mutations at positions S218, K223, and R225 exhibited significantly impaired G-protein coupling to multiple different Gα isoforms when compared to the wild-type US28-NP construct (Fig. 4). The attenuation of G-protein coupling for all mutants was not as severe as complete alanine substitution of the DRY motif located in the second intracellular loop, and no significant difference was observed regarding mutation at position S220 (Fig. 4). Interestingly, despite observing a non-significant difference in scintillation proximity assays, we observed a decrease in the ability of the K223 mutant to couple with Gα_q_ isoforms. Together, these data indicate that mutation at specific residues within the ICL3 region of US28 results in an inability to efficiently couple with respective G-proteins, consequently attenuating US28 signaling activity.

### Mutations in HCMV US28 ICL3 fail to efficiently reactivate from latent infection in CD34^+^ HPCs

**Fig 5:**
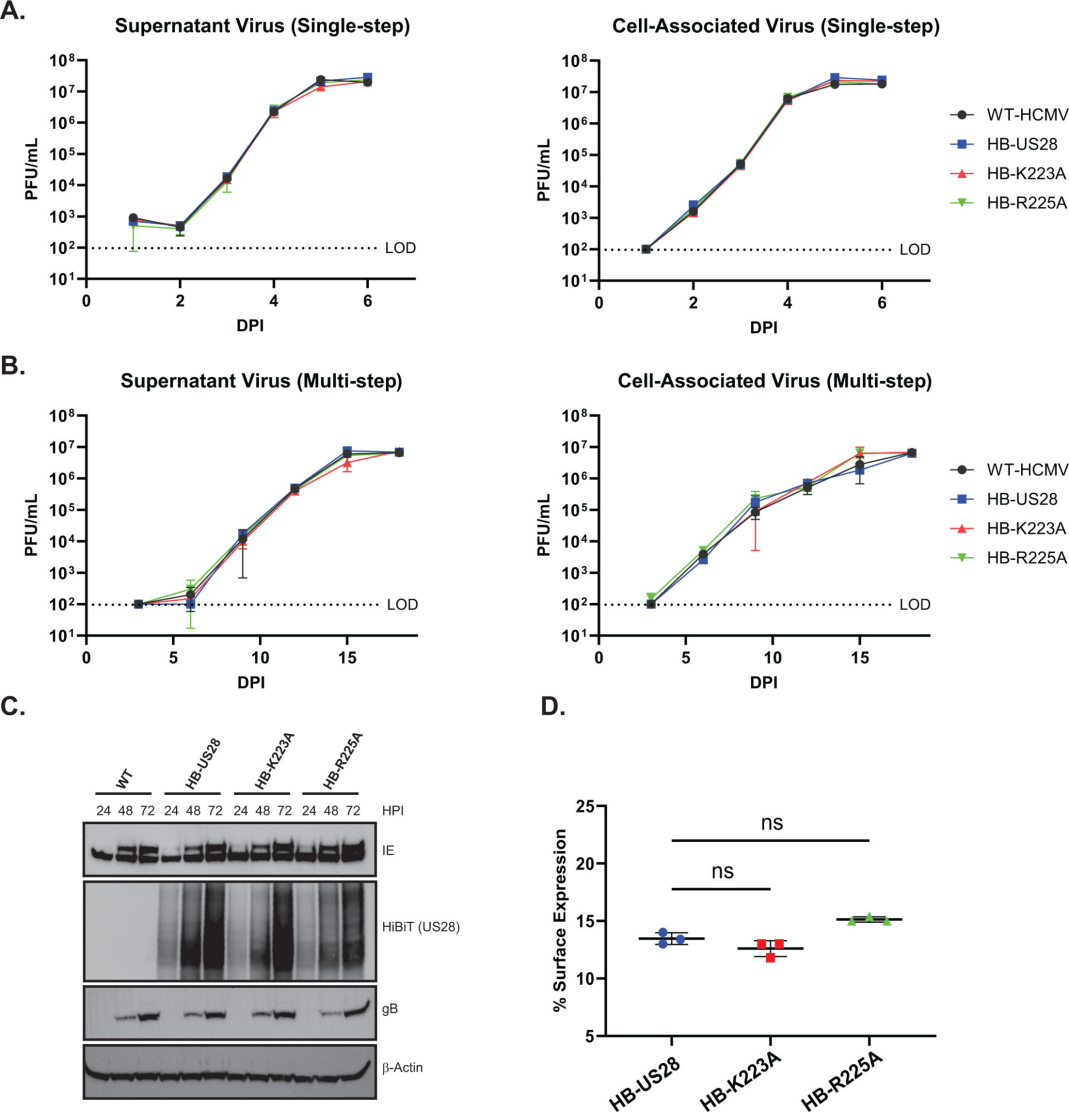
Characterization of US28 ICL3 recombinant viruses. NHDF cells were infected with the indicated viruses at a multiplicity of infection (MOI) of (**A**) 3 or (**B**) 0.01. Supernatant and cell-associated virus were harvested at the indicated timepoints post-infection and titered using confluent monolayers of NHDFs. Error bars represent the standard error of the mean between biological triplicates. (**C**). NHDFs were infected with the indicated viruses at an MOI of 2 and lysates were harvested at the indicated time points post-infection. The presence of indicated proteins was confirmed *via* traditional immunoblot using the indicated primary antibodies or the Nano-Glo HiBiT Blotting System (Promega). Representative blot shown from triplicate experiments. (**D**) NHDFs were infected with the indicated HiBiT-US28 tagged viruses at an MOI of 2. At 3 days post-infection, total and surface expression was assessed using the Nano-Glo HiBiT Lytic and Extracellular Detection Systems (Promega). Error bars represent the standard error of the mean between triplicate experiments. Statistical significance was calculated by one-way ANOVA followed by Dunnett’s multiple comparison post hoc analysis.

While transient transfection in established cell lines is a reliable and tractable model for studying the effects of viral chemokine signaling in isolation, the complexities of viral infection are not accurately captured in these systems. To monitor the effects of US28-ICL3 mutations within the context of infection, we generated recombinant viruses using the HCMV TB40/E-GFP backbone and engineering the HiBiT tag onto the N′ terminus of US28 followed by alanine substitutions at positions K223 and R225 (TB40/E-GFP-HB-US28, TB40/E-GFP-HB-US28-K223A, and TB40/E-GFP-HB-US28-R225A), as both of these mutations, when compared to wild-type US28, have reduced signaling and G-protein-coupling activities but retain expression and localization. To determine the growth kinetics of our recombinant viruses, we performed both single and multistep growth analysis in primary human fibroblasts. All three recombinant viruses exhibited similar replication kinetics to the parental virus confirming previous reports that US28 is not required for viral replication in fibroblasts (Fig. 5A and B) (20, 32, 66, 67). Moreover, recombinant viruses exhibited similar immediate early, early, and late viral protein expression, including US28, at multiple time points post-infection (Fig. 5C). To assess whether point mutations alter US28 localization within the context of infection, we performed HiBiT surface vs total expression assays in infected human fibroblasts. Consistent with localization and internalization experiments conducted in transiently transfected cells (Fig. 3; Fig. S4), no detectable difference in surface expression was observed between the three recombinant viruses indicating that mutations within the ICL3 region do not significantly affect subcellular localization of US28 (Fig. 5D).

**Fig 6:**
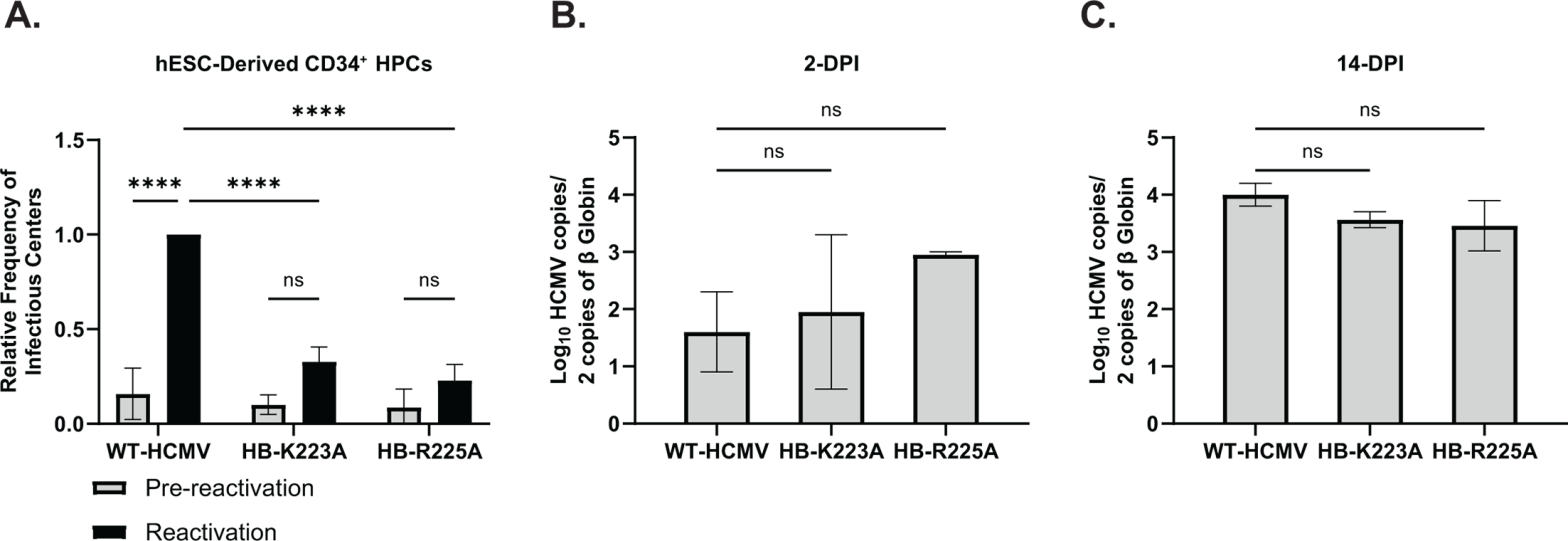
US28 ICL3 mutants fail to efficiently reactivate from latent infection. (**A**) hESC-derived CD34^+^ HPCs were infected with HCMV TB40/E-GFP (WT-HCMV), HCMV TB40/E-GFP-HB-US28-K223A (HB-K223A), and/or HCMV TB40/E-GFP-HB-US28-R225A (HB-R225A) at a multiplicity of infection (MOI) of 3 for 48 hours. Cells were FACS isolated for viable, CD34^+^ /GFP^+^ HPCs and were cultured above a stromal cell support layer for 12 days to establish latent infection. At 14 DPI, half of the cells were treated with a reactivation cocktail and plated onto a fibroblast monolayer, the other half of the cells were lysed and used to infect fibroblasts directly (pre-reactivation control). Reactivation was assessed by the frequency of infectious centers as determined *via* ELDA (51, 52) at 3 weeks post-plating. Data are shown as fold change in infectious centers, as compared to the WT reactivation group, for triplicate experiments. Error bars represent the standard error of the mean and statistical significance was calculated using two-way ANOVA followed by Tukey’s post hoc analysis (****P < 0.0001)*.* (**B**) At 2-DPI and (**C**) 14-DPI, total DNA was harvested from infected CD34^+^ HPCs and viral genomes were quantified *via* qPCR using primers and probes specific for the viral UL141 gene. Viral genomes were normalized to total cell number using human β-globin as a reference gene. Data are shown as the mean Log_10_ transformed values for triplicate experiments. Error bars represent the standard error of the mean and statistical significance was calculated by one-way ANOVA followed by Dunnett’s multiple comparison post hoc analysis.

Since US28-mediated signaling plays a major role in the establishment of viral latency and the capacity to reactivate (35, 37, 38), we hypothesized that US28-ICL3 recombinant viruses with impaired G-protein coupling may exhibit an inability to establish latent infection or deficiencies in their ability to reactivate. To test this hypothesis, we infected both primary and hESC-derived CD34^+^ HPCs with HCMV TB40/E-GFP, HCMV TB40/E-GFP-HB-US28-K223A (HCMV HB-K223A), and/or HCMV TB40/E-GFP-HB-US28-R225A (HCMV HB-R225A) (49, 50). Infected CD34^+^ HPCs were isolated *via* FACS and cultured above a murine stromal support layer under conditions that favor latent infection. At 14-DPI, the frequency of infectious centers was assessed *via* ELDA, as previously described (48, 50). In both primary and hESC-derived CD34^+^ HPCs infected with HCMV HB-K223A or HCMV HB-R225A, we observed deficits in the ability of the virus to efficiently reactivate from latent infection when compared with wild-type-infected cells (Fig. 6A; Fig. S7). To assess whether loss of genomes could explain the reactivation defect observed with either of the recombinant viruses containing ICL3 mutations, we quantified viral genome copies from infected hESC-derived CD34^+^ HPCs at the beginning and end of latent infection *via* quantitative PCR. A comparable number of viral genomes were present in cells infected with WT-HCMV and recombinant viruses with mutations in the ICL3 region of US28, suggesting a reactivation defect occurs with these mutants (Fig. 6B and C). Together, these results indicate that US28 G-protein coupling and downstream signaling play an integral role in the viral reactivation process in CD34^+^ HPCs.

### US28 third intracellular loop mutations affect viral reactivation *in vivo*

**Fig 7:**
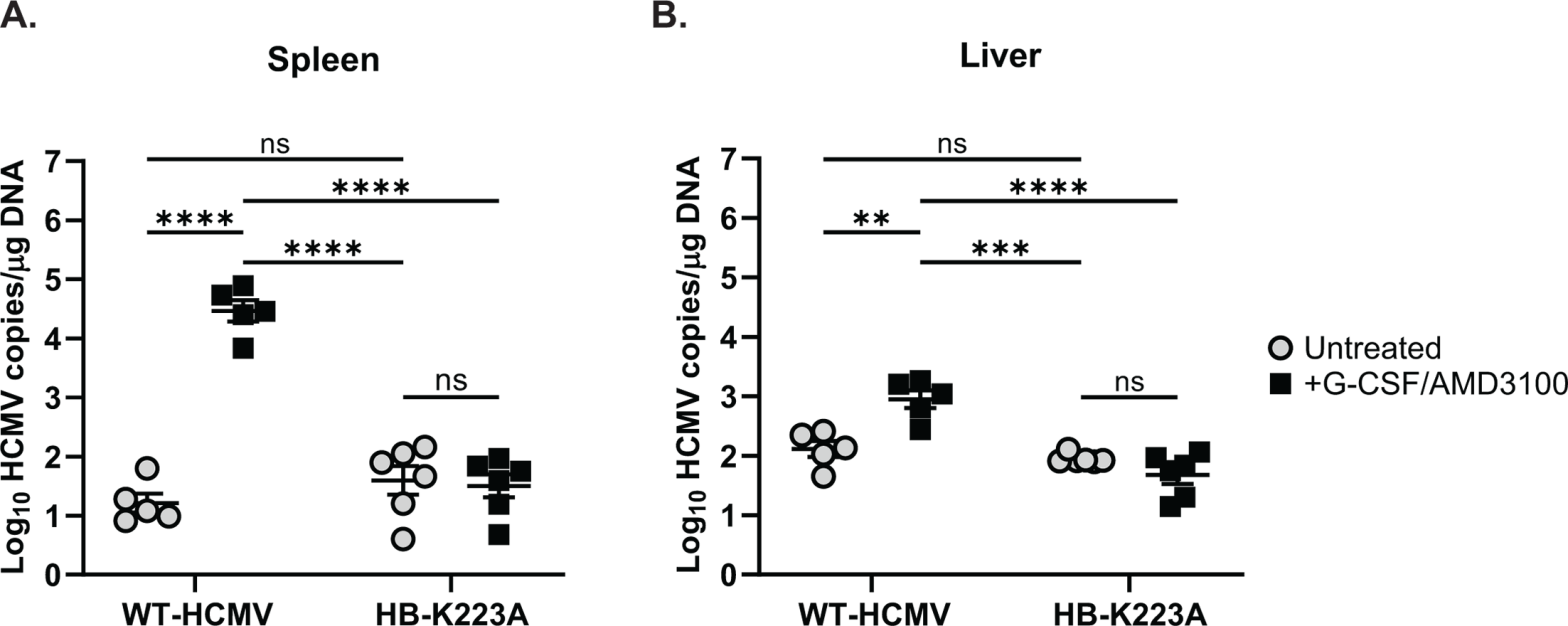
US28 ICL3 mutants fail to efficiently reactivate from latent infection in humanized mice. Humanized NSG (huNSG) mice were infected *via* intraperitoneal injection of NHDFs infected with HCMV TB40/E-GFP (WT-HCMV) or HCMV TB40/E-GFP-HB-US28-K223A (HB-K223A) (35, 68). At 4 weeks post-infection, half of the mice in each infection group were treated with G-CSF and AMD3100 to induce viral reactivation. Control, latently infected mice were treated with DMSO. At 1-week post-treatment, mice were euthanized prior to harvesting (**A**) spleen and (**B**) liver tissues. Total DNA was extracted using DNAzol, and HCMV viral load was determined by qPCR from two tissue sections per mouse. Error bars represent the standard error of the mean between the average DNA copies per huNSG mouse (*n*=5 mice per group). All samples were compared by two-way ANOVA followed by Tukey’s multiple comparisons test between experimental groups (**P < 0.01/***P < 0.001/****P < 0.0001)*.*

To further examine the impact of attenuating US28 G-protein interactions on viral reactivation, we utilized a humanized NOD-scid IL2Rγc^null^ (huNSG) mouse model of HCMV infection (68). In these experiments, huNSG mice received engraftment of cord blood-derived CD34^+^ HPCs and, at 5 weeks post-engraftment, were infected with HCMV TB40/E-GFP (WT-HCMV) or HCMV TB40/E HB-K223A by intraperitoneal injection of infected fibroblasts (68). At 4 weeks post-infection, half of the latently infected huNSG mice were treated with a reactivation cocktail consisting of G-CSF and the CXCR4 antagonist AMD3100 to induce cellular differentiation and mobilization. One week post-mobilization, spleen and liver tissue were harvested from all animals, and viral genome copies, indicative of viral reactivation, were assessed *via* quantitative PCR probing. Comparable viral loads in liver and spleen tissues were observed for animals latently infected with HCMV HB-K223A and WT-HCMV (Fig. 7A and B). This finding demonstrates that HCMV HB-K223A can establish latency in humanized mice to WT levels, mirroring our findings in CD34^+^ HPCs. By contrast, animals infected with HCMV HB-K223A that received reactivation stimulus showed a significant reduction in viral genome copies in both liver and spleen tissues compared to WT-HCMV-infected animals (Fig. 7A and B), indicating that the virus has a reactivation defect *in vivo*. Together, these results confirm that protein-protein interactions mediated by the third intracellular loop of US28 are not required for the establishment or maintenance of latent infection but are required for HCMV reactivation both *in vitro* and *in vivo*.

## DISCUSSION

While a growing number of studies have examined signal transduction through the viral chemokine receptor US28, the specific mechanism by which US28 initiates these signaling cascades remains unclear. In the present report, we conduct a mutational analysis of the third intracellular loop (ICL3) of US28 to identify residues that are integral for G-protein coupling, downstream signaling, and viral reactivation. Our results indicate that alanine substitutions at positions S218, K223, and R225 significantly attenuate US28-mediated activation of mitogen-activated protein (MAP) kinase and RhoA signal transduction pathways. Furthermore, we show that mutations at positions S218, K223, and R225 result in impaired coupling to multiple Gα isoforms, including Gα_i/o_, Gα_q/11_, and Gα_12/13_, despite maintaining normal surface expression and internalization kinetics. Utilizing primary- and hESC-derived CD34^+^ HPCs, we show that recombinant viruses with mutations in the ICL3 region of US28 fail to efficiently reactivate from latent infection. These results were further confirmed in a humanized mouse model of HCMV infection where recombinant viruses established and maintained latent infection but did not reactivate post-mobilization. Taken together, our results identify novel residues within the ICL3 region of US28 that are required for G-protein coupling, downstream signaling activity, and viral reactivation from latency in hematopoietic progenitor cells.

Mounting evidence into the mechanism of rhodopsin-like receptor coupling to cognate Gα subunits indicates that specific residues at the base of TM3 and TM6 (ICL2/3) are major determinants of G-protein coupling and specificity (54–58, 69). For instance, a recent study examining autoregulation of the β_2_ adrenergic receptor (β_2_AR) found that residues within the third intracellular loop participate in ionic interactions to stabilize weakly coupled G-proteins allowing them to efficiently interface with the β_2_AR receptor (56). Consistent with these findings, our results indicate that mutation of polar and charged residues within the ICL3 region of US28 results in deficient G-protein coupling and downstream signaling activity. We show that the observed reduction in G-protein coupling and signaling are independent of subcellular localization as receptors harboring mutations in the ICL3 region exhibit normal internalization kinetics (Fig. 3). Interestingly, we did not observe any significant difference in signal transduction or G-protein coupling with regard mutation at position S220 (Fig. 2 and 4). This result would suggest that this residue may be occluded from interacting with heterotrimeric G-protein complexes and not participate in coupling but may have additional functional purposes. Furthermore, we note that the K223 residue is important for US28 coupling to Gα_q/11_ isoforms, but we observe no effect with this mutant in IP_3_ accumulation experiments (Fig. 2 and 4). We surmise that this may be due to differences in assay sensitivity and/or residual activity as we did not observe a complete loss of coupling activity. The observed deficit in G-protein coupling for other US28 ICL3 mutant constructs was not as severe as results obtained by mutation of the canonical G-protein coupling domain located in the second intracellular loop, and no specific effect was observed when examining different Gα isoforms (Fig. 4). These results would suggest that residues within the ICL3 region of US28 may mediate secondary interactions with G-proteins irrespective of classification, or that they are required for stabilizing the active conformation of US28. These hypotheses are not without basis as a recent publication authored by De Groof et al. showed similar attenuation of G-protein coupling utilizing intrabodies recognizing the ICL3 region of US28 (70). Results reported here confirm these findings and suggest that the ICL3 region is integral for US28–G-protein interactions. Further structural and biochemical research examining the constitutive and ligand-induced signal transduction will be required to delineate the specific role of this region in relation to signal transduction.

Despite being dispensable for viral replication in fibroblasts (Fig. 5), the functional consequences of US28 downstream signaling are cell-type specific, with differential effects observed dependent on the infected cell type. Importantly, previous work indicates that US28 is expressed during both latent and lytic phases of the viral lifecycle and that US28 is required for both the maintenance of latent infection and the capacity to reactivate (19, 35–38, 71). Recently, our group published a study describing US28-mediated activation of RhoA to enhance emergence out of latent infection and that pharmacologic inhibition of US28-mediated RhoA signaling blocks viral reactivation *in vitro* and *in vivo* (39). Similarly, results reported here validate that US28 signaling activity is required for viral reactivation *in vitro* and *in vivo* and identify novel motifs required for US28 signaling activity. Despite showing that Gα_q/11_ signal transduction is important for viral reactivation (Fig. 1) and that mutation of K223 results in no significant change in IP_3_ accumulation (Fig. 2), we observed a deficit in the ability of this virus to reactivate from latent infection *in vitro* and *in vivo* (Fig. 6 and 7). Moreover, none of the mutations within the ICL3 region of US28 completely abrogated coupling to heterotrimeric G-protein complexes (Fig. 4) yet still exhibited an inability to reactivate from latent infection. These results suggest that, in addition to stimulating PCL-β activity, US28 is modulating viral latency and reactivation through multiple signal transduction pathways concurrently and that these signaling events are not all or nothing per se. Therefore, it is likely that a receptor as promiscuous as US28 serves a multipurpose function in regard to HCMV latency and reactivation, acting as a rheostat for the fine-tuning of cell signaling networks altering the cellular microenvironment in favor of the virus.

Contradictory to previous well-conducted studies regarding the requirement of US28 to maintain latent infection within cells of myeloid lineage (19, 36, 72), we did not observe any deficits in the ability of our recombinant viruses to establish or maintain a latent infection. We suggest that incongruencies reported here could be due to differences in cell source or recombinant virus construction. Previous studies have utilized recombinant viruses lacking the complete US28 ORF while viruses used here are only deficient in receptor function. Moreover, CD34^+^ HPCs represent a heterogeneous population of cells with distinct differences dependent on the source from which they are harvested (49, 73, 74). While we made use of hESC- and primary fetal liver-derived HPCs in this study, cord blood-derived CD34^+^ HPCs likely have subtle genetic and morphological differences which may explain inconsistencies in results reported elsewhere. An in-depth comparison of different HCMV latency models would certainly be of great value to the field but is nonetheless outside the scope of the current study.

The γ-herpesviruses, Epstein Barr Virus (EBV) and Kaposi Sarcoma Herpesvirus (KSHV), encode their respective viral GPCRs: BILF1 and ORF74, respectively. Despite exhibiting a low degree of sequence similarity to US28, the ICL3 region of these vGPCRs contains several polar and charged residues that have been implicated to be important for G-protein coupling (75–77). EBV-BILF1 and KSHV-ORF74 are important for immune evasion, modulation of cellular proliferation, transformation, and regulation of cellular signal transduction (78–82). Results reported here provide insights into the functional importance of the ICL3 region of these vGPCRs and have highlighted its potential as a therapeutic target for developing novel antiviral strategies aimed at disrupting viral GPCR signaling pathways. Future research focusing on the detailed molecular interactions within the ICL3 region of EBV-BILF1 and KSHV-ORF74 will be crucial for unraveling the precise mechanisms underlying viral pathogenesis and for informing the development of targeted therapies against gamma herpesvirus-associated diseases.

US28 is notable for its ability to interact with multiple Gα isoforms and initiate diverse signaling cascades, influencing various cellular processes crucial for viral pathogenesis. Because of its substantial role in viral latency and reactivation, there has been interest in developing novel therapeutics targeting US28. To date, these candidate therapeutics encompass multiple different mechanisms of action including small molecule antagonists and inverse agonists, fusion toxin-protein molecular trojan horse chimeras, and single domain nanobodies targeting US28 (83). One such US28-specific intrabody, VUN103, was shown to bind the second and third intracellular loops of US28 to displace Gα_q_ and prevent constitutive US28-mediated activation of STAT3, NF-κB, and NFAT (70). Consistent with these findings, results reported here indicate that the third intracellular loop of US28 is a major determinant of G-protein coupling and is required for downstream signaling. To our knowledge, this is the first study to investigate the ICL3 region of US28 as pertaining to viral latency and reactivation. Our findings will guide future studies aiming to develop novel therapeutics against HCMV-associated disease.

## MATERIALS AND METHODS

### Plasmids

US28-NP was generated by cloning the Natural Peptide (NP) tag (GVTGWRLCERILA) in-frame with the C-term of US28. HB-US28 was generated by cloning the HiBiT (HB) tag (VSGWRLFKKIS) in-frame with the N-term of US28. Primers used for generating plasmid constructs are listed in Table S1. Fragments were PCR amplified and cloned into the pcDNA3.1-vector. Mutations in the third intracellular loop of US28 were generated by site-directed mutagenesis, substituting alanine for the indicated amino acid using the Q5 Mutagenesis Kit (NEB) following the manufacturer’s recommended procedure. All constructs were confirmed by sequencing and transformed into TOP10 *Escherichia coli* cells (Invitrogen). Large-BiT tagged Gα subunits were kindly provided by Julien Hanson (Addgene plasmid ID: 134359, 134360, 134364, and 134363) (63). Reporter plasmids pRL-SV40 Renilla luciferase (Rluc), pGL4.33[luc2P/SRE/Hygro], and pGL4.34[luc2P/SRF-RE/Hygro] containing SRE and SRF responsive elements driving luciferase expression were purchased from Promega.

### Cells and virus

Normal human dermal fibroblasts (NHDFs) were obtained from ATCC (PCS-201–010), and human embryonic kidney (HEK)-293 cells were obtained from Microbix. Both cell types were maintained in Dulbecco’s modified Eagle’s medium (DMEM), 10% FBS, streptomycin, penicillin, and glutamine at 37°C and 5% CO_2_. M2-10B4 and S1/S1 stromal cells were obtained from Stem Cell Technologies and cultured as previously described (50). WA01 human embryonic stem cells (hESCs) were obtained from the WiCell Research Institute—National Stem Cell Bank and were cultured as previously described (48, 84). The HCMV strain TB40/E-GFP, which constitutively expresses green fluorescent protein under the SV40 promoter (85), was amplified in NHDFs as previously described (86–88). Recombinant US28 mutant viruses were generated using a two-step recombineering procedure (51) utilizing the HCMV TB40/E-GFP bacterial artificial chromosome (BAC) (85). Viral constructs were confirmed by next-generation sequencing prior to plaque purification and clonal expansion. Viral titers were determined *via* plaque assay on NHDF cells and aliquots stored at −80°C. For viral growth analyses, single-step growth curves were carried out at a multiplicity of infection (MOI) of 3.0 PFU/mL, and multi-step growth curves were carried out at an MOI of 0.01 PFU/mL. Supernatant and cell-associated viruses were harvested at multiple time points post-infection and titered *via* limiting dilution plaque assay on NHDF cells.

### Immunoblot

Cell lysates were harvested using RIPA Lysis Buffer (Santa-Cruz Biotechnology) supplemented with HALT protease inhibitor (Thermo-Fisher Scientific). Proteins were separated on a 4%–12% SDS-PAGE gel and transferred onto PVDF membranes. Immunoblots were performed using antibodies directed against β-Actin (sc-47778, Santa-Cruz Biotechnology), HCMV IE1/IE2 (MAB8131, Millipore-Sigma), HA (sc-7392, Santa-Cruz Biotechnology), HCMV gB (sc-69742, Santa-Cruz Biotechnology), P-p44/42 MAPK T202/Y204 (4370S, Cell Signaling Technology), p44/42 MAPK (9102S, Cell Signaling Technology), Rho (1862332, Thermo Scientific), and, if required, the appropriate HRP-conjugated secondary antibody (sc-525409). HiBiT-tagged proteins were visualized using the Nano-Glo HiBiT Blotting System (Promega).

### Cytotoxicity assay

Compound cytotoxicity was determined using the CellTiter-Glo kit (Promega) (89). Briefly, NHDF cells were seeded into treated black 96-well plates at a density of 1.5 × 10^4^ cells per well. The following day, cells were treated with a compound at the indicated concentration or an equivalent amount of DMSO (vehicle) in triplicate. At 72 hours post-treatment, cellular cytotoxicity was assessed by adding 100 µL of assay reagent to each well and incubating for 10 minutes with agitation per the manufacturer’s recommendation. Luminescence was measured using a Promega GloMax Navigator luminometer. Well luminescence, indicative of the number of living cells per well, was converted to percent cell viability in Microsoft Excel by dividing luminescence values in experimental wells by the value in control wells containing untreated cells and multiplying by 100. Values obtained were used to calculate 50% cellular cytotoxicity (CC_50_) by nonlinear regression analysis within GraphPad Prism 10.0 software.

### HCMV-gHnLUC viral replication assay

To assess the effects of the indicated compounds on viral replication, we used our TB40/E-gHnLUC reporter virus assay as previously described (39). Briefly, NHDF cells were seeded into treated black 96-well plates at a density of 1.5 × 10^4^ cells per well. The following day, cells were treated with compound at the indicated concentration or an equivalent amount of DMSO (vehicle) in triplicate and were infected with TB40/E-gHnLUC at a multiplicity of infection (MOI) of 0.3. At 72 hours post-infection, viral replication was assessed using the Nano-Glo Luciferase Assay kit (Promega) as previously described (39). Luminescence was measured using a Promega GloMax Navigator luminometer. Well luminescence, indicative of viral growth, was converted into percent inhibition in Microsoft Excel by dividing luminescence values in experimental wells by the value in control wells containing untreated cells and multiplying by 100. The resulting values were used to calculate the 50% inhibitory concentration (IC_50_) by nonlinear regression within GraphPad Prism 10.0 software.

### Luciferase reporter assay

Reporter assays were conducted as previously described (90) using the Dual Luciferase Assay Kit (Promega). Briefly, HEK-293 cells were seeded into treated black 96-well plates at a density of 3.0 × 10^4^ cells per well. The following day, cells were co-transfected in triplicate with the indicated US28 construct and/or the empty pcDNA3.1 vector, the indicated FireFly luciferase reporter element, and the Renilla luciferase reporter element using Fugene4K (Promega) following the manufacturer’s recommended protocol. At 18 hours post-transfection, the growth medium was replaced with serum-free DMEM with or without small-molecule inhibitors at the indicated concentrations. At 6 hours post-media replacement, the cell culture medium was removed and 20 µL of Passive Lysis Buffer (Promega) was added to each well. Plates were incubated for 20 minutes with agitation at room temperature. Luciferase assay reagents were reconstituted and 100 µL was injected per well in a Promega GloMax Navigator luminometer. Assay results were transferred to a Microsoft Excel spreadsheet, normalized to Renilla luciferase, and analyzed using GraphPad Prism 10.0 software.

### Scintillation proximity assay

IP_3_ accumulation assays were conducted as previously described (91). Briefly, HEK-293 cells were seeded into 96-well plates pre-treated with poly-D-lysine at a density of 3.5 × 10^4^ cells per well. The following day, cells were transfected in duplicate with 20 ng of the indicated US28 construct and/or the empty pcDNA3.1 vector using Lipofectamine 2000 (Thermo-Fisher Scientific). At 24 hours post-transfection, the culture medium was aspirated and replaced with 0.1 mL media supplemented with 0.5 μL of 1 mCi/mL [3H]myo-inositol. Following 24 hours of incubation, cells were treated as follows: the medium was aspirated, and wells were washed twice with Hanks’ Balanced Salt Solution (HBSS, Gibco). 100 µL of HBSS buffer containing 10 mM LiCl was added to each well and incubated for 90 minutes at 37°C. After incubation, the plates were put on ice, the medium was aspirated and 40 μL of 10 mM formic acid was added to each well to lyse the cells. The [^3^H]IPs in the formic acid cell lysates were thereafter quantified by Ysi-poly-D-Lys-coated SPA beads Briefly, 35 μL of lysate were transferred to a new white 96-well plate and mixed with 60 μL of SPA-YSi (PerkinElmer) bead solution (12.5 mg/mL) Plates were sealed, agitated for at least 30 minutes and centrifuged. SPA beads were allowed to settle and react with the extract for 8 hours before radioactivity was determined using a MicroBeta2 (2450–0060, Perkin Elmer). All determinations were made in duplicate.

### FRET-based real-time internalization assay

The internalization kinetics of wild-type and ICL3 US28 mutant constructs were assessed as previously described (92). Briefly, HEK-293 cells were transiently transfected with the indicated constructs and/or the empty vector (SNAP-FRT) using Lipofectamine 2000 (Thermo-Fisher Scientific) according to the manufacturer’s recommendation. At 48 hours post-transfection, cells were incubated with Tag-Lite SNAP-Lumi4Tb (donor) (Cisbio, SSNPTBD) for 1 hour at 4°C. Afterward, cells were washed four times with HBSS. Following washing, 20 µL of reconstituted fluorescein‐O′‐acetic acid (acceptor) (Sigma‐Aldrich, catalog no. 88596) was added to each well. Internalization was measured for 80 minutes utilizing a PerkinElmer EnVision 2104 Multilabel Reader using the following settings: 340 nm (excitation), 520 nm (acceptor), and 615 nm (donor). Results are presented as the baseline corrected ratio of donor over acceptor emissions (615/520 nm). To compare internalization across constructs, the area under the curve (AUC) was calculated as described previously (93). Assay results were plotted in GraphPad Prism 10.0 software.

### Live cell G-protein coupling assay

US28 ICL3 mutant G-protein coupling was assessed as previously described (63) using the Nano-Glo Live Cell Assay system (Promega). Briefly, HEK-293 cells were seeded into treated black 96-well plates at a density of 3.0 × 10^4^ cells per well. The following day, cells were co-transfected in triplicate with a 1:1 ratio of the indicated US28-NP construct and/or the empty pcDNA3.1 vector, and Large BiT linked Gα subunit using Fugene4K (Promega) following the manufacturer’s recommended procedure. At 18 hours post-transfection, the growth medium was replaced with Opti-MEM media (Thermo Fisher Scientific). At 6 hours post-media replacement, 25 µL of reconstituted Nano-Glo Live Cell assay reagent was added to each well, and plates were briefly incubated with agitation. Luminescence, indicative of US28–G-protein coupling, was measured using a Promega GloMax Navigator luminometer. Assay results were transferred to a Microsoft Excel spreadsheet, backgrounds subtracted and plotted in GraphPad Prism 10.0 software.

### HiBiT surface expression assay

NHDF or HEK-293 cells were seeded into cell culture-treated black 96-well plates at a density of 1.5 × 10^4^ cells per well. The following day, triplicate wells were infected with the indicated HiBiT-tagged US28 recombinant viruses at an MOI of 1.0, or transfected with the indicated HiBiT-tagged US28 pcDNA3.1(-) constructs. At 72 hours post-infection or 24 hours post-transfection, surface vs total HiBiT expression was evaluated using the Nano-Glo HiBiT Extracellular and Lytic Detection kits (Promega) following the manufacturer’s recommended procedure. Luminescence was measured using a Promega GloMax Navigator luminometer. Assay results were transferred to a Microsoft Excel spreadsheet, background subtracted, normalized to the HiBiT control protein, and % surface expression was determined using the ratio of extracellular vs lytic luminescence. Results were analyzed using GraphPad Prism 10.0 software.

### Active Rho pulldown and detection assay

GST-Rhotekin pulldown was performed as previously described (39). Briefly, HEK-293 cells were seeded into treated 10 cm dishes at a density of 3.0 × 10^5^ cells/mL. The following day, cells were co-transfected with the indicated US28 constructs or the empty pcDNA3.1 vector using Fugene4K (Promega) following the manufacturer’s recommended protocol. At 24 hours post-transfection, the growth medium was replaced with serum-free DMEM, and cells were cultured for an additional 24 hours. Cells were washed once with PBS and lysed in a buffer containing 25 mM Tris-HCl, 150 mM NaCl, 5 mM MgCl_2_, 1% NP-40, and 5% glycerol. Lysates were clarified by centrifugation at 4°C and protein concentrations were normalized using the Qubit Protein BR Assay Kit (Invitrogen). Proteins were isolated using the Active Rho Pulldown and Detection kit (Thermo Scientific) according to the manufacturer’s recommendations. The total amount of active RhoA was assessed *via* immunoblot using the indicated primary antibodies. Quantification shows the relative expression of RhoA bound to GTP normalized against the loading control (beta-actin) and compared to transfection with the wild-type US28 receptor.

### HCMV latency and reactivation assay

hESC-derived CD34^+^ HPCs were differentiated from WA01 human embryonic stem cells using the commercial STEMdiff Heme feeder-free hematopoietic differentiation kit (Stem Cell Technologies) as previously described (48, 49, 94). Primary CD34^+^ HPCs were isolated from deidentified donors using magnetic bead separation (Miltenyi Biotech) as previously described (35, 95). HPCs were cultured in SFEMII with 10% BIT serum replacement, stem cell cytokines (stem cell factor, FLT3L, IL-3, and IL-6 [PeproTech]), and penicillin/streptomycin as previously described (35, 48–50, 90, 95). CD34^+^ HPCs were infected with the indicated viruses at an MOI of 2 for 48 hours prior to isolation by fluorescence-activated cell sorting (FACS) using a FACSAria (BD FACS Aria equipped with 488, 633, and 405 nm lasers, running FACS DIVA software) to obtain a pure population of viable GFP^+^, CD34^+^, HPCs as previously described (50, 95). Infected cells were co-cultured in transwell culture dishes above monolayers of irradiated M2-10B4 and S1/S1 stromal cells. At 14 days post-infection, HPCs were serially diluted in RPMI-1640 medium containing 20% FBS, 2 mM L-glutamine, 100 U/mL penicillin, 100 µg/mL streptomycin, 15 ng/mL granulocyte-colony stimulating factor (G-CSF), and 15 ng/mL granulocyte-macrophage colony-stimulating factor (GM-CSF) and overlaid onto confluent monolayers of NHDFs cultured in 96-well plates. To quantify the levels of pre-reactivation infectious virus, a fraction of the HPC cultures were mechanically disrupted and lysates were serially diluted and then added to NHDFs cultured in 96-well plates. Cell cultures were microscopically visualized for the presence of GFP^+^ weekly, for up to 4 weeks, to assess the reactivation frequency from latently infected cells and the presence of preformed infectious virus by extreme limiting dilution assay (50, 52).

### HCMV infection of humanized mice

Mouse procedures were performed in accordance with approved Institutional Animal Care and Use Committee (IACUC) protocol 0922 under the recommendations of the American Association for Accreditation of Laboratory Animal Care (AAALAC). Mice were housed in the Vaccine & Gene Therapy Institute at Oregon Health & Science University vivarium using microisolator cages and fed sterile food and water ad litem. Both sexes of animals were used. Humanized mice were generated by irradiating NOD.Cg-PrkdcscidIL2Rγtm1Wjl/SzJ (NSG) mice (Jackson Laboratories) by sublethal irradiation of 7- to 10-week-old mice (250 cGy by 137Cs γ-irradiation). The irradiated animals were reconstituted by tail vein injection of 1 × 10^5^ human cord blood-derived CD34^+^ HPCs as described previously (68). At 4 weeks post-engraftment, mice were distributed to experimental groups normalized for engraftment success as determined by the percentage of human CD45^+^ lymphocytes in the periphery. Humanized mice were dosed with 1 mL of 4% thioglycolate (Brewer’s medium; BD) by intraperitoneal (i.p.) injection and then injected i.p. with approximately 10^5^ PFU of cell-associated virus per mouse with the indicated viruses. At 4 weeks post-infection, the animals were divided into two groups. One group of latently infected mice were treated with 100 µL of Neupogen (G-CSF; 300  mg/mL; Amgen) by subcutaneous pump and 125 µg of AMD3100 administered by i.p. injection to mobilize progenitor cells and promote HCMV reactivation (35, 96). At 1 week post-mobilization, the mice were euthanized *via* CO_2_ administration according to AAALAC euthanasia guidelines, and then blood, bone marrow, spleen, and liver tissues were collected for further analysis.

### Viral DNA quantification

Primers and probes recognizing HCMV UL141 were used to quantify viral genomes by quantitative real-time PCR (35). Briefly, total DNA was extracted from portions of the mouse spleen and liver using DNAzol (ThermoFisher) according to the manufacturer’s recommendations. Dilutions of purified HCMV BAC DNA were used to create a standard curve. A 1 µg sample of total DNA was added to each reaction well of TaqMan FastAdvance PCR master mix (Applied Biosystems) and samples were analyzed in triplicate on a StepOnePlus TaqMan PCR machine (Applied Biosystems) with an initial activation at 50°C for 2  minutes and 95°C for 20 s, followed by 40 cycles of 1 s at 95°C and 20 s at 60°C. TaqMan results were analyzed using ABI StepOne software and graphed using GraphPad Prism 10.0 software.

## SUPPLEMENTAL MATERIAL

Supplemental material jvi.01801-24-s0001.docx Figures S1 to S7; Table S1.

ASM does not own the copyrights to Supplemental Material that may be linked to, or accessed through, an article. The authors have granted ASM a non-exclusive, world-wide license to publish the Supplemental Material files. Please contact the corresponding author directly for reuse.
