# Supplementary material for: Third intracellular loop of HCMV US28 is necessary for signaling and viral reactivation
Source: J Virol. 2024 Dec 10;99(1):e01801-24. doi: 10.1128/jvi.01801-24 (PMC11784217; doi:10.1128/jvi.01801-24)
Supplement: Supplemental material — Figures S1 to S7; Table S1. [file jvi.01801-24-s0001.docx]

**Supplemental Material: Third Intracellular Loop of HCMV US28 Is Necessary for Signaling and Viral Reactivation**

Samuel Medica^a,b^, Michael Denton^a^**,** Nicole L. Diggins^a^, Olivia Kramer-Hansen^c^, Lindsey B. Crawford^a,^*, Adam T. Mayo^a^, Wilma D. Perez^a^, Michael A. Daily^a^, Christopher J. Parkins^a^, Luke E. Slind^a^, Lydia J. Pung^a^, Whitney C. Weber^a,b^, Hannah K. Jaeger^a^, Zachary J. Streblow^a^, Gauthami Sulgey^a^, Craig N. Kreklywich^a^, Timothy Alexander^a^, Mette M. Rosenkilde^c^, Patrizia Caposio^a^, Meaghan H. Hancock^a^, & Daniel N. Streblow^a,b,d,#^

^a^ Vaccine and Gene Therapy Institute, Oregon Health and Science University, Beaverton, Oregon, USA

^b^ Department of Molecular Microbiology and Immunology, Oregon Health and Science University, Portland, Oregon, USA

^c^ Department of Biomedical Sciences Molecular Pharmacology, University of Copenhagen, Copenhagen, Denmark

^d^ Division of Pathobiology and Immunology, Oregon National Primate Research Center, Beaverton, Oregon, USA

* Current affiliation: Department of Biochemistry, University of Nebraska – Lincoln, Lincoln, Nebraska, USA

**Running Title: Mutational Analysis of HCMV US28 ICL3**

^#^Address correspondence to Daniel N. Streblow, [streblow@ohsu.edu](mailto:streblow@ohsu.edu)


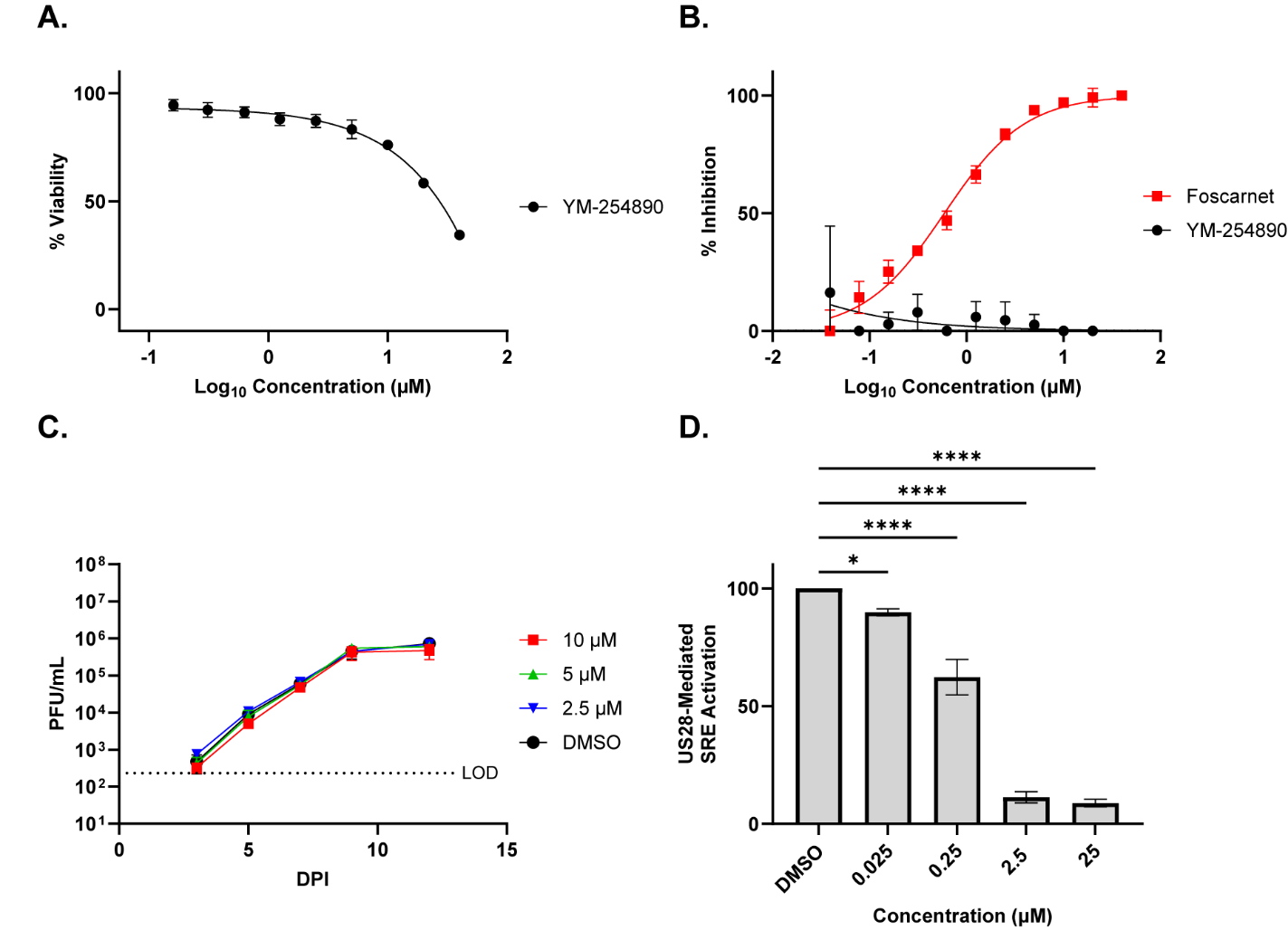


**Supplemental Figure 1: Characterization of the Gα_q/11_ Inhibitor YM-254890.** **(A)** NHDFs were treated with the indicated concentration of YM-254890 or an equivalent amount of DMSO (vehicle). At 3 days post-treatment, cellular viability was assessed using the CellTiter-Glo assay system (Promega). Concentrations of YM-254890 were Log_10_ transformed, and data is plotted as percent viability relative to the cells treated with vehicle alone. Error bars represent the standard error of the mean between triplicate experiments. **(B)** NHDFs were treated with the indicated concentrations of YM-254890, Foscarnet, or an equivalent amount of DMSO (vehicle) followed by infection with TB40/E-gHnLUC at a MOI of 0.3. At 3 days post-infection, luminescence was quantified using the Nano-Glo luciferase assay system (Promega). Concentrations of the indicated compounds were Log_10_ transformed and plotted as percent inhibition relative to the cells treated with vehicle alone. Error bars are representative of the standard error of the mean between triplicate experiments. **(C)** NHDFs were treated with the indicated concentration of YM-254890 and infected with the TB40/E-GFP (WT-HCMV) at a MOI of 0.01. Supernatant virus was harvested at the indicated timepoints post-infection and tittered over an NHDF monolayer. Error bars represent the standard error of the mean between biological duplicates. **(D)** HEK-293 cells were transfected with HiBiT tagged US28 or the empty pcDNA3.1 vector along with Renilla and SRE reporter plasmids. At 18 hours post-transfection, the media was changed to serum-free DMEM supplemented with the indicated concentrations of YM-254890. Luciferase activity was measured using the Dual-Luciferase Reporter assay system (Promega) 6 hours post-media replacement. Error bars represent the standard error of the mean between triplicate experiments. Statistical significance was calculated by one-way ANOVA followed by Dunnett’s multiple comparisons test between experimental groups *(*p < 0.05 / ****p < 0.0001).*


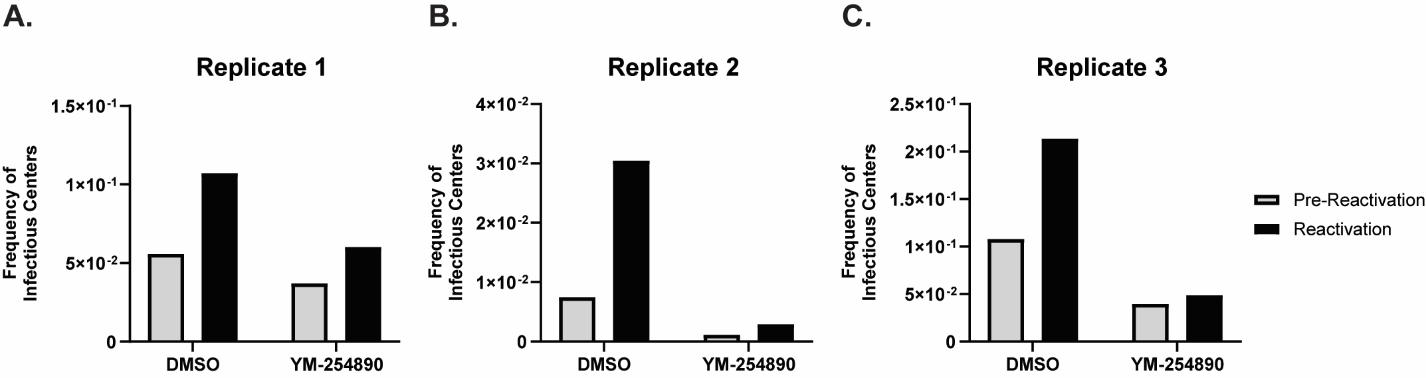


**Supplemental Figure 2: Replicate Experiments for YM-254890 Latency Assays.** hESC-derived CD34^+^ HPCs were infected with TB40/E-GFP at a MOI of 2 for 48 hours. Cells were FACS isolated for viable CD34^+^/GFP^+^ HPCs and were cultured above a murine stromal cell support layer for 12 days to establish latent infection. Cells were treated with 1 µM YM-254890 (Gα_q/11_ inhibitor) or an equivalent amount of DMSO throughout the latency culture. **(A – C)** At 14-DPI, half of the cells were treated with reactivation cocktail and plated onto a fibroblast monolayer, the other half of the cells were lysed and used to infect fibroblasts directly (pre-reactivation control). Reactivation was assessed by the frequency of infectious centers as determined via ELDA (87,95) at 3 weeks post-plating.


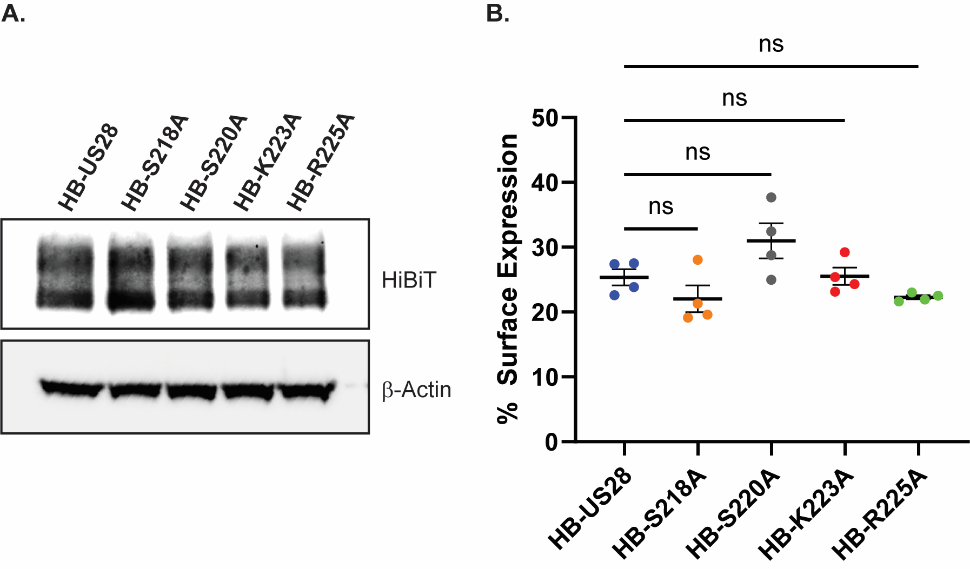


**Supplemental Figure 3: HiBiT Tagged US28 ICL3 Mutant Constructs are Efficiently Expressed.** **(A)** HEK-293 cells were transfected with the indicated HiBiT-tagged US28 constructs. Expression was confirmed via immunoblot using the indicated antibodies and the Nano-Glo HiBiT Blotting System (Promega) on whole lysates harvested 48 hours post-transfection. Representative blot shown from triplicate experiments. **(B)** HEK-293 cells were transfected with the indicated HiBiT-US28 tagged constructs or the empty vector. At 24 hours post-transfection, total and surface expression was assessed using the Nano-Glo HiBiT Lytic and Extracellular Detection Systems (Promega). Error bars represent the standard error of the mean between triplicate experiments. Statistical significance was calculated by one-way ANOVA followed by Dunnett’s multiple comparison post-hoc analysis.


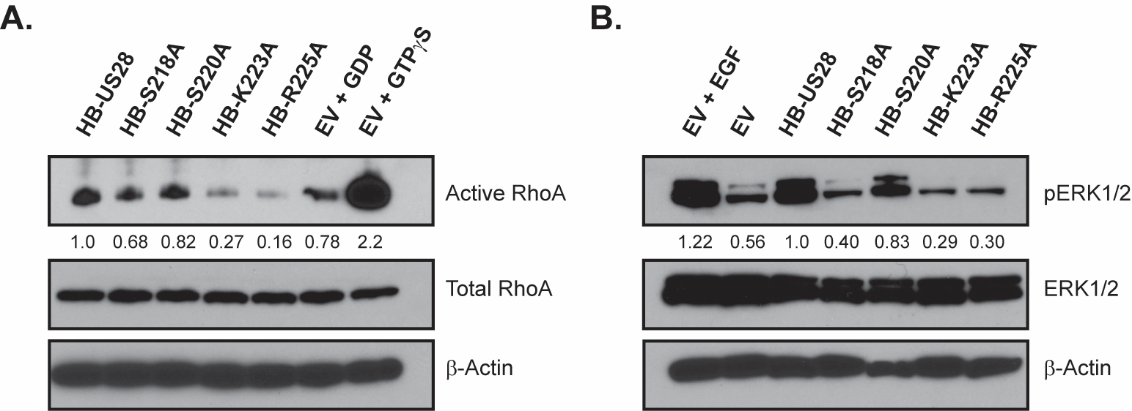


**Supplemental Figure 4: HiBiT Tagged US28 ICL3 Mutant Constructs Exhibit Attenuated MAPK and RhoA Signal Transduction. (A)** HEK-293 cells were transfected with the indicated HiBiT-tagged US28 constructs or the empty vector. At 24 hours post-transfection, cell culture media was exchanged to serum-free DMEM for an additional 24 hours prior to harvesting whole cell lysates. Lysates were subjected to GST-Rhotekin pulldown kit according to the manufacturer’s (ThermoFisher) recommendations. RhoA activation was assessed via immunoblot using the indicated primary antibodies. Quantification shows the relative expression of RhoA bound to GTP normalized against β-actin and set relative to transfection with the wild type US28 receptor. Representative blot shown from triplicate experiments. **(B)** HEK-293 cells were transfected with the indicated HiBiT-tagged US28 constructs or the empty vector. At 24 hours post-transfection, cell culture media was exchanged to serum-free DMEM for an additional 24 hours prior to harvesting whole cell lysates. ERK phosphorylation was assessed via immunoblot using the indicated primary antibodies. Quantification shows the relative expression of phosphorylated ERK normalized against β-actin and set relative to transfection with the wild type US28 receptor. Representative blot shown from triplicate experiments.


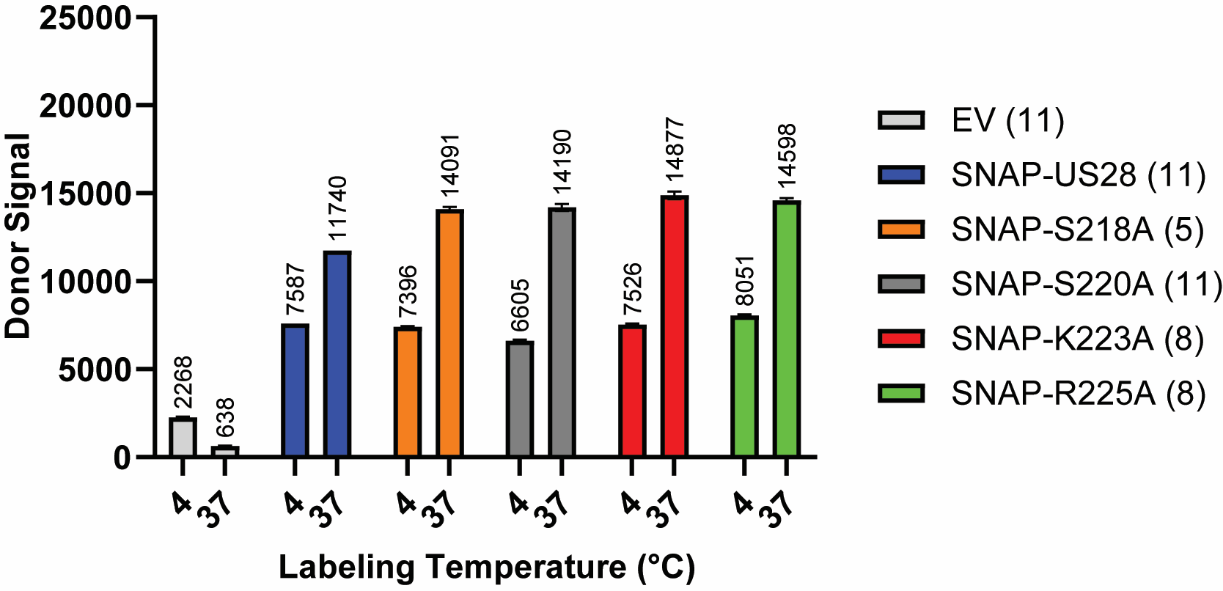


**Supplemental Figure 5: Optimization of SNAP-tagged US28 Construct Transfection Conditions.** HEK-293 cells were transfected with the indicated SNAP-US28 constructs or the empty vector (EV). At 48 hours post-transfection, cells were treated with SNAP-Lumi4-Tb (donor) for one hour prior to washing. Fluorescein was added to the appropriate wells and internalization kinetics were measured for 80 minutes. Data is plotted as the baseline corrected donor signal intenstity.

**
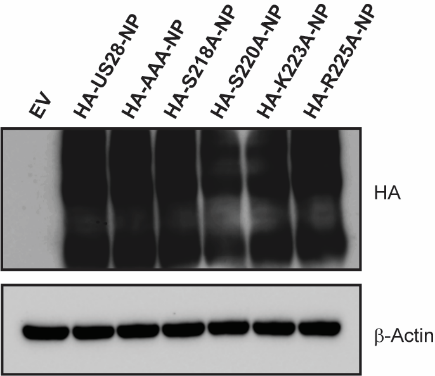
**

**Supplemental Figure 6: Natural Peptide Tagged US28 ICL3 Mutant Constructs are Efficiently Expressed.** HEK-293 cells were transfected with the indicated HA-tagged US28 constructs. Expression was confirmed via immunoblot using the indicated antibodies on whole lysates harvested at 48 hours post-transfection. Representative blot shown from triplicate experiments.


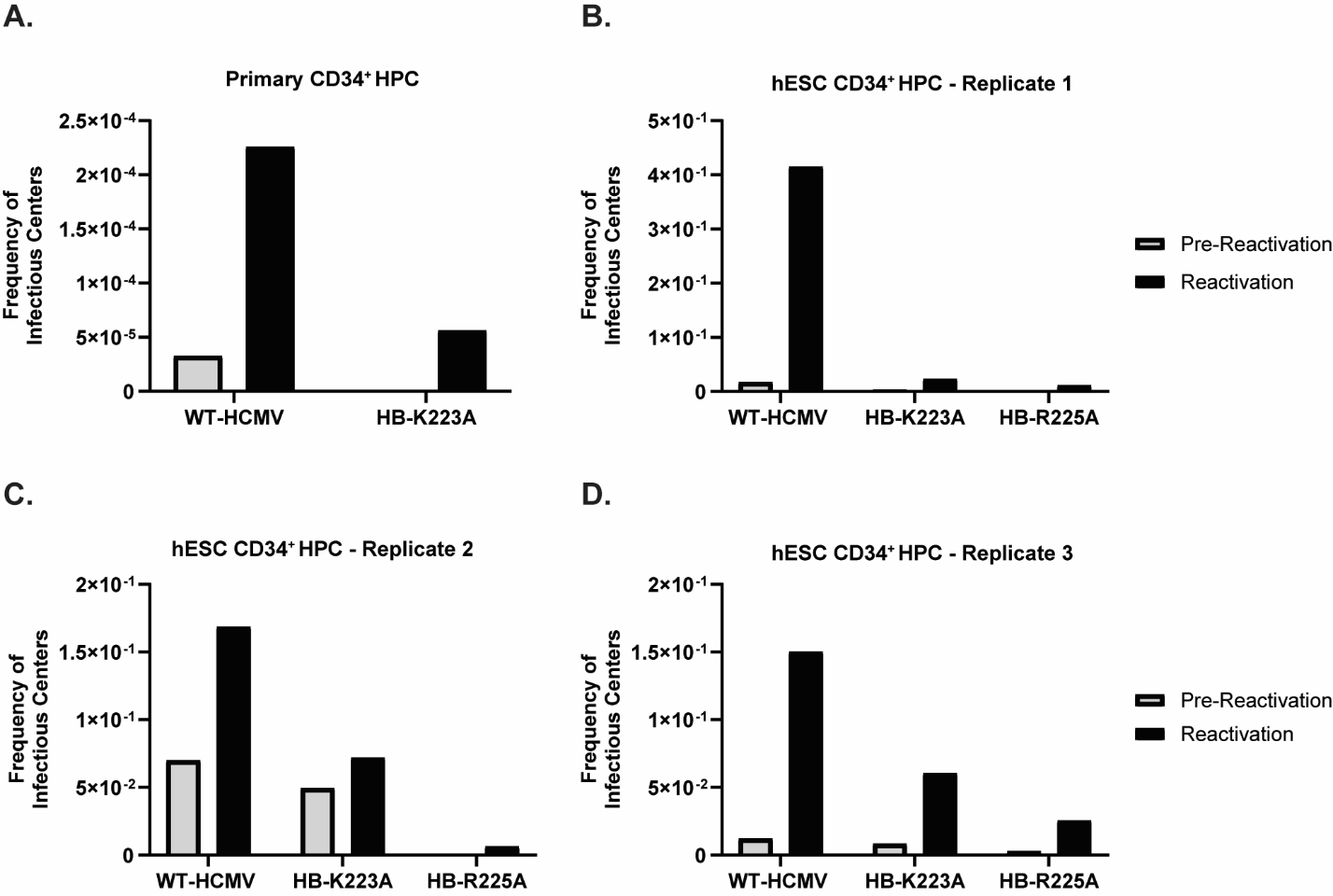


**Supplemental Figure 7: Replicate Experiments for US28-ICL3 Latency Assays (A)** Primary or hESC-derived **(B – D)** CD34^+^ HPCs were infected with HCMV TB40/E-GFP (WT-HCMV), HCMV TB40/E-GFP-HB-US28-K223A (HB-K223A), and/or HCMV TB40/E-GFP-HB-US28-R225A (HB-R225A) at a MOI of 3 **(A)** or 2 **(B – D)** for 48 hours. Cells were FACS isolated for viable, CD34^+^ /GFP^+^ HPCs and were cultured above a stromal cell support layer for 12 days to establish latent infection. At 14-DPI, half of the cells were treated with reactivation cocktail and plated onto a fibroblast monolayer, the other half of the cells were lysed and used to infect fibroblasts directly (pre-reactivation control). Reactivation was assessed by the frequency of infectious centers as determined via ELDA (87,95) at 3 weeks post-plating.

| **Target** | **Designation** | **Sequence** |
| --- | --- | --- |
| US28-Natural Peptide | Forward | GTCGCGTCTCACAAATTATACCGGGCGGCATGAAGACGATCATCGC |
|  | Reverse | GCGATGATCGTCTTCATGCCGCCCGGTATAATTTGTGAGACGCGAC |
| HiBiT-US28 | Forward | GAATTCATGGTGAGCGGCTGGCGGCTGTTCAAGAAGATTAGCACACCGACGACGACGACCGC |
|  | Reverse | AAGCTTCGGTATAATTTGTGAGACGCGACACGCCTCG |
| US28-S218A | Forward | AGAATCGTTGCGGTGGCTCAGTCGCGCCACA |
|  | Reverse | TGTGGCGCGACTGAGCCACCGCAACGATTCT |
| US28-S220A | Forward | CGTACAATGCGGCCTGCGTGGCGCGACTGAGA |
|  | Reverse | GGCCTTTGTGGCGCGCCTGAGACACCGCAAC |
| US28-K223A | Forward | TCTCAGTCGCGCCACGCAGGCCGCATTGTACG |
|  | Reverse | CGTACAATGCGGCCTGCGTGGCGCGACTGAGA |
| US28-R225A | Forward | TCGCGCCACAAAGGCGCCATTGTACGGGTACT |
|  | Reverse | AGTACCCGTACAATGGCGCCTTTGTGGCGCGA |
| US28-AAA | Forward | CGGAGATTGCACTCGCAGCCGCCTACGCTATTGTTTACATGAGATATC |
|  | Reverse | GATATCTCATGTAAACAATAGCGTAGGCGGCTGCGAGTGCAATCTCCG |

**Supplemental Table 1:** Primers used for generating plasmid constructs. Sequences are shown from the 5’ to 3’ orientation.
